# Preventive and Therapeutic Effects of *Punica granatum* L. Polyphenols in Neurological Conditions

**DOI:** 10.3390/ijms24031856

**Published:** 2023-01-17

**Authors:** Simona Aleksandrova, Ralitza Alexova, Stela Dragomanova, Reni Kalfin, Ferdinando Nicoletti, Paolo Fagone, Maria Cristina Petralia, Katia Mangano, Lyubka Tancheva

**Affiliations:** 1 Department of Biological Activity of Natural and Synthetic Substances, Institute of Neurobiology, Bulgarian Academy of Sciences, 1113 Sofia, Bulgaria; 2 Department of Medical Chemistry and Biochemistry, Medical Faculty, Medical University—Sofia, 2 Zdrave St., 1431 Sofia, Bulgaria; 3 Department of Pharmacology, Toxicology and Pharmacotherapy, Faculty of Pharmacy, Medical University, 9002 Varna, Bulgaria; 4 Department of Health Care, South-West University “Neofit Rilski”, Ivan Mihailov St. 66, 2700 Blagoevgrad, Bulgaria; 5 Department of Biomedical and Biotechnological Sciences, University of Catania, Via S. Sofia 89, 95123 Catania, Italy; 6 Department of Clinical and Experimental Medicine, University of Messina, 98122 Messina, Italy

**Keywords:** pomegranate, punicalagin, ellagic acid, mental health, antioxidants

## Abstract

Pomegranate (*Punica granatum* L.) is a polyphenol-rich food and medicinal plant containing flavonols, anthocyanins, and tannins. Ellagitannins (ETs) are the most abundant polyphenols in pomegranate. A growing body of research shows that polyphenol-rich pomegranate extracts and their metabolites target multiple types of brain cell and support their redox balance, proliferation and survival, as well as cell signaling. Independent studies have demonstrated that the significant neuroprotective effects of ETs are mediated by their antioxidant and anti-inflammatory effects, their chelating properties, by their ability to activate various signaling pathways, as well as the ability to influence mitochondrial damage, thus regulating autophagy, apoptosis and neurotransmitter signaling. The multitude of in vitro and in vivo studies summarized in the present review suggest that pomegranate polyphenols act on both neuronal and glial cells directly, and also affect blood–brain barrier function, restoring redox balance in the blood and brain and increasing blood flow to the brain.

## 1. Pomegranate and Ellagitannins

Pomegranate (*Punica granatum* L.) is a polyphenol-rich food and medicinal plant containing flavanols, anthocyanins, and tannins.

Polyphenols are part of the human diet, as they are found in a variety of fruits, vegetables, and grains. These plant metabolites have long been used in the traditional medicine of many cultures. According to the free-radical theory, polyphenolic compounds exert their beneficial effects by binding and neutralizing pathogenetic free radicals, forming stable compounds. Insufficient dietary intake of antioxidants is associated with an increased risk of chronic pathological conditions including cancer, metabolic diseases, and neurodegenerative diseases. In addition, dysregulated oxidative responses have also been implicated in the pathogenesis of neurodegenerative diseases (NDD), and several polyphenols have been investigated in connection with these diseases [1,2,3].

Accordingly, polyphenols seem to provide health benefits in these diseases [4,5]. As a consequence, in recent years, the interest in the pharmacological activity and therapeutic potential of polyphenols has continued to grow.

Pomegranate has been used for hundreds of years in traditional Chinese, Ayurveda and Persian medicine, for the treatment of atherosclerosis, diabetes, hypertension, hyperlipidemia, and some types of cancer, as well as for ulcer and diseases of the oral cavity. To this day, pomegranate and its extracts (juice, fruit peel extract, fruit, seeds, oil, etc.) are highly represented in the traditional medicine and cuisine of Eastern cultures. Along with other superfoods such as turmeric, ginger and nutmeg, when used as nutraceuticals, they show pluripotency and an excellent safety profile [6,7].

The brilliant red color of the ripe pomegranate fruit is given by the anthocyanins, while the yellow color of the husk is provided by a class of hydrolyzable tannins, the ellagitannins (ETs) [8,9]. ETs are large molecules composed of Ellagic acid (EA) or multiple units of it, condensed to a carbohydrate or a sugar alcohol core, usually a glucose molecule. More than 60 pomegranate ETs have been characterized [10]. ETs can be extracted during commercial juice preparation, and comprise more than 70% of the extracted polyphenols [11]. While not unique to pomegranate, the ETs reach higher concentrations in *P. granatum* than in other fruit, berries, and nuts [10,12,13]. In 180 mL of pomegranate juice there are approximately 318 mg of punicalagin (PUN) isomers, 12 mg of ellagic acid (EA), and 75 mg of other hydrolyzable tannins [14]. Other non-edible tissues of the plant, such as the bark, roots, leaves, and flowers are also used in traditional medicine and contain different composition of ETs, such as punicacortein, corilagin, granatins, free EA, and much less PUN [10,11,14].

**Figure 1:**
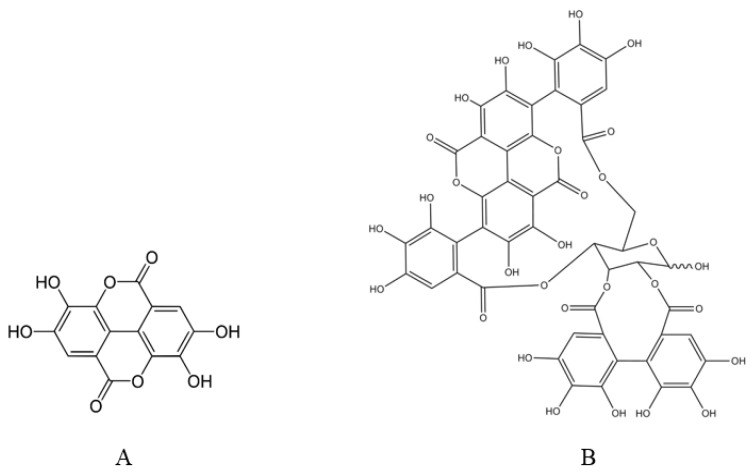
Chemical structure of Ellagic acid (**A**) and Punicalagin (**B**).

Not only are the ETs the most abundant polyphenols in pomegranate, they are also the component contributing the most to the antioxidant capacity of the plant [15,16], which is several fold higher than the antioxidant activity of red wine, green tea, or orange juice, although it varies between pomegranate accessions [9]. A much more significant source of variability is the plant part from which the extract is produced. In the juice from obtained from the arils, the antioxidant activity correlates with the content of anthocyanin. However, whole fruit homogenate is twenty times more potent as antioxidant, which correlates better with the hydrolyzable tannin content and the PUN concentration [9]. In pomegranate peel, PUN represents up to 85% (*w*/*w*) of the polyphenol content and is 500 times more concentrated than EA [9,10] (Figure 1).

Due to this variability, many studies have used purified compounds, rather than polyphenol-rich pomegranate extracts.

**Figure 2:**
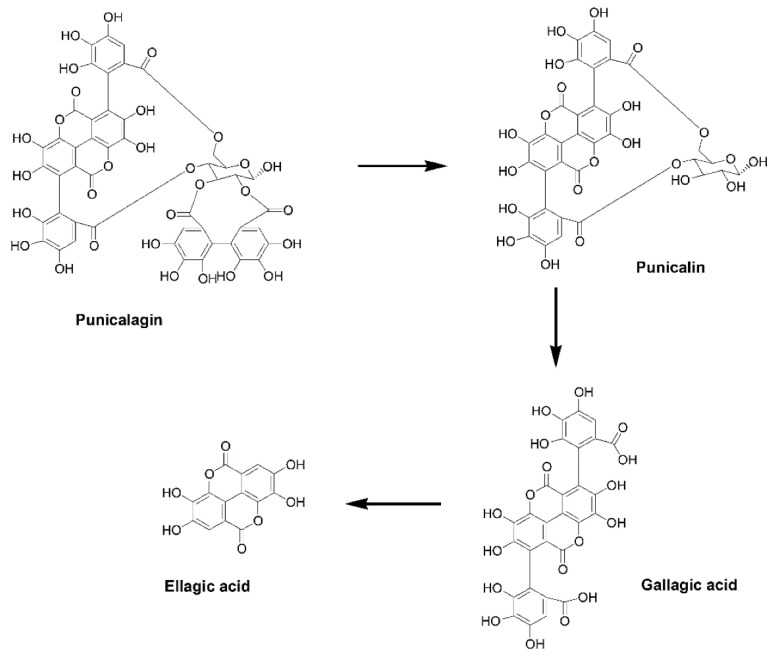
Natural production of ellagic acid through biodegradation of punicalagin.

The most abundant ET is PUN, which exists as alpha and beta isomers in nearly equal proportions. Upon hydrolysis, one PUN molecule gives a total of three EA molecules, via the intermediates punicalin and gallagic acid [17,18,19] (Figure 2).

EA (2,3,7,8-tetrahydroxy-chromeno [5,4,3-cde]chromene-5,10-dione) is the aglycone component of the ellagitanins—a group of the hydrolyzable tannins [10]. It is a major metabolite of ellagitannins, after oral administration. EA is an amphiphilic molecule with hydrophilic, as well as lipophilic domains. It is poorly soluble in water, but dissolves relatively well in organic solvents, such as methanol and dimethyl sulphoxide. For pharmaceutical use, the best solvents are N-methyl-2-pyrrolidone, which increases transdermal penetration, polyethylene glycol 400, for parenteral formulations, and triethanolamine, for injections and topical formulations [20].

Due to its solubility in organic solvents, EA serves well as a lipophilic antioxidant, which together with its ability to scavenge peroxide radicals makes it an excellent candidate as inhibitor of chain oxidation processes (e.g., lipid peroxidation) [21,22]. The most important factors contributing to the antioxidant capacity of EA are its ability to inhibit lipid peroxidation, even at micromolar concentration, as well as its ability to scavenge free radicals. EA scavenges ROS and RNS (reactive oxygen species and reactive nitrogen species) such as hydroxyl radicals, peroxyl radicals, ·NO_2_ radicals, as well as peroxynitrite, and its antioxidant capacity is similar to that of other well-known powerful antioxidants, such as vitamin C and vitamin E [23,24,25].

## 2. Central Nervous System (CNS) as a Therapeutic Target for Pomegranate Polyphenols

The brain is a complex system, and its homeostasis relies on the interaction and integration of several cell types, neurons, the supporting glia, and the endothelial component of the blood–brain barrier (BBB). In addition, during inflammation, the resident cell populations may become enriched by peripheral immune cells. All of these cells may be both drivers and targets of the inflammatory and oxidative damage associated with central nervous system (CNS) and mental health deterioration [26].

A growing body of research shows that polyphenol-rich pomegranate extracts and their metabolites target multiple types of brain cell and support their redox balance, proliferation and survival, as well as cell signaling. A substantial number of studies have focused on the mechanisms by which ETs affect NDD onset and disease progression. Nevertheless, the ability of pomegranate ETs to control oxidative stress and regulate the immune response in the context of mental health support has been much less explored. Table 1 summarizes the pomegranate products currently used in research studies.

### 2.1. Effect of ETs on Symptoms of Anxiety and Depression

Overall, the polyphenols reduced anxious and depressive behavior when administered over a wide time frame, ranging from 30 min before behavior testing, to several weeks of chronic application. EA showed anxiolytic activity in mice after both single and chronic oral administration [27]. The effect persisted after repeated treatment, indicating that mice did not develop tolerance to EA [27]. In rats, EA and PUN were effective at doses <0.1 mg/kg and higher concentrations did not have beneficial effect, perhaps due to prooxidant effects starting to take place [28].

Sleep deprivation in mice can resemble chronic stress, causing memory impairment and anxiety, with high levels of proinflammatory cytokines, such as Interleukin (IL)-1β, IL-6 and Tumor Necrosis Factor (TNF)-α and oxidative stress, as determined by the evaluation of the superoxide dismutase, glutathione peroxidase and malonyl dialdehyde levels. EA pretreatment for 2 weeks not only improved the memory and learning deficits, induced by sleep deprivation, but also reduced the associated anxiety [29]. On the other hand, direct application of PUN to the brain via the intracerebroventricular route did not improve the memory deficits [30]. The two ETs therefore appear to exert different effects on chronically stressed rodent brains. Other studies have also shown that depressive behaviors, induced by chronic stress, improved after supplementation with either pomegranate peel extract or EA [31,32].

Our previous studies demonstrated significant beneficial effects of EA in Institute of Cancer Research (ICR) mice in two experimental in vivo models of oxidative stress—influenza infection and dementia induced by scopolamine [33]. EA administration clearly improved the learning capability in both healthy control mice as well as in mice exposed to systemic oxidative stress. A stronger effect was observed in animals with influenza virus infection compared to healthy controls. A high rate of memory protection (50%) in the scopolamine-treated group was also observed. The potential mechanisms that facilitate the EA effect on cognitive function may occur through dopamine modulation in the brain as well as a direct antioxidant effect [33].

At the biochemical level, the results showed lower levels of lipid peroxidation compared to the controls. The EA molecule is amphiphilic, and this allows good cell membrane permeability, as well as the ability to interact with membranes, by forming weak chemical bonds or embedding within the membrane structures, thus providing a protective antioxidant shield [34]. In female mice with aluminum-induced memory impairment, long-term supplementation with pomegranate juice ameliorated their memory performance [35]. In a similar model of aluminum-induced stress in male mice, the severity of anxiety and depression was reduced by treatment with pomegranate juice. The stress-reducing features of pomegranate juice correlated with lower cortisol levels in plasma. Exposure to copper nanoparticles also led to neuronal damage accompanied by anxious and depressive-like behavior in rats, which were also improved in animals receiving pomegranate juice.

Both per os (p.o.) and intraperitoneal (i.p.) administration of EA reduced depressive behavior in rodents, and the treatment improved the swimming behavior (that seems to depend on the involvement of the serotonergic system), more than the climbing behavior (related to the noradrenergic system) [27,36,37]. In the same study, PUN affected climbing behavior and not swimming behavior [38]. In other experiments, an aqueous pomegranate extract improved immobility and swimming, but not the climbing behavior, despite the use of a similar setup and doses used by Cervantes-Anaya et al. [38]. This is surprising, given that PUN is the predominant ET in pomegranate extracts. The discrepancy may be due to the cited content of EA in the extract used in that study, which was 3-fold higher than that of PUN [38]. The different responses to EA and PUN further support that both polyphenols are able to interact with the nervous system, but exert their effects via different mechanisms. As the predominant effect of the pomegranate extract may be determined by its specific polyphenol composition, there is the strong need for the standardization of the pomegranate extracts, when reproducible nutraceutical effects are sought after.

As neither EA nor PUN affect locomotor function, they are considered to be specific to anxiety and/or depressive behavior, and not to be a general CNS stimulant [27,28,37,38,39]. In addition, they may have a synergistic effect with established therapeutic agents, as a combination of aqueous pomegranate extract with citalopram showed a higher antidepressant effect [40].

### 2.2. Antinociceptive Action of Ellagitannins

Neuropathic pain can emerge after central sensitization. It can be resistant to treatment and last for long periods of time, becoming a significant burden for mental health. As oxidative stress is implicated in its pathogenesis, it may represent a suitable therapeutic target to compounds, such as pomegranate ETs [41,42,43]. A comparison of two pomegranate varieties showed that their peel extracts affected pain perception, both centrally and peripherally, and the results depended on the plant variety [41]. Pomegranate fruit extract administered p.o. also reduced hyperalgesia in neuropathic pain in rats. Purified PUN applied intrathecally prevented hyperalgesia to cold and mechanical stimuli, as well as by formalin-induced inflammation in rats. The effect on both early and late stages of formalin-induced pain suggested that prostaglandins, as well as opioid system, were involved, and both pomegranate extracts and purified PUN had anti-inflammatory activities in addition to the analgesic action [41,42,43].

### 2.3. Effects of ETs on Learning and Memory

Accumulating damage from chronic or acute inflammation and oxidative stress may also be implicated in the process of ageing, NDD, mood disorders, and cognitive decline. A systemic pro-inflammatory and oxidative environment associated with acute or chronic emotional stress or with metabolic disorders like diabetes can further precipitate neuroinflammation and oxidative stress in the brain.

Dietary supplementation of pregnant mice with pomegranate juice protected against neurodegeneration in newborn mice subjected to hypoxic-ischemic brain injury [36]. The results revealed that polyphenolic substances from pomegranate juice were found in the plasma of young mice whose mothers drank pomegranate juice.

Memory dysfunction after cardiac surgery is a common problem and may be related to a diffuse ischemic state, caused by microemboli dislodging during the procedure. Convincing results were obtained in a pilot clinical trial in patients undergoing cardiac surgery. Patients undergoing coronary artery and/or valve bypass surgery received 2 g of pomegranate extract or placebo daily from one week before surgery to 6 weeks after surgery [44]. The evaluation was performed on the basis of a set of neuropsychological tests for memory evaluation 1 week before surgery (baseline), 2 weeks after surgery, and 6 weeks after surgery. The placebo group, as expected, showed a significant memory deficit after surgery, while the experimental group showed a preventive effect in terms of memory dysfunction, and this effect lasted for at least 6 weeks after surgery.

Recent data reported by Shen et al. [45] shows that UA can have a beneficial effect also in Multiple Sclerosis (MS). In fact, its administration in a mouse model of the disease, both in the early and in advanced stages, leads to a reduction of autoimmune responses, with a reduction in the number of dendritic cells, Th17-helper lymphocytes, and macrophages. Reduced levels of pro-inflammatory mediators and suppression of the demyelination processes were also observed [45].

In different rodent models of Alzheimer’s Disease (AD) (transgenic model of familial AD and Aβ1–42 intracerebroventricular injection model), pomegranate extract improved behavioral performance and decreased amyloid accumulation in the hippocampus. The mechanism of action involves suppressing the deposition of toxic amyloid and improving its clearance [46,47].

The multifactorial neuroprotective effect of pomegranate was observed in a study of performed using a mice model of chronically infused amyloid beta neurodegeneration. The authors reported that consumption of pomegranate peel extract resulted in reduced amyloid plaque density, increased brain-derived neurotrophic factor (BDNF) secretion and decreased acetylcholinesterase (AChE) activity. In addition, decreased lipid peroxidation and concentration of the pro-inflammatory cytokine TNF-α have been reported [48].

In humans, the effects of pomegranate juice on memory disorders were investigated in a randomized, placebo-controlled, double-blind trial in elderly people with impaired memory. Approximately 250 mL (8 oz) of pomegranate juice taken daily for one month was found to improve verbal memory and neural activity during a visual memory task. After 4 weeks, the pomegranate group also showed significant improvement in verbal memory. Additionally, compared to the placebo group, the pomegranate group had increased functional magnetic resonance imaging (fMRI) activity during verbal and visual memory tasks. Although preliminary, these results suggest the role of pomegranate juice in enhancing memory function by increasing task-related functional brain activity. The fMRI results suggest that the mechanisms may involve a task-related increase in cerebral blood flow, particularly during associative encoding tasks. Future studies are needed to validate these results in larger samples and determine the long-term effects of pomegranate juice on a range of comprehensive cognitive functions [49].

In the amyloid beta (Aβ1–42)-overexpressing AD model, UA improves associative memory [50]. Administration of UA in a transgenic mouse model of AD led to improved learning ability, improved memory, and reduced neuronal death, as well as neurogenesis in the hippocampus. UA decreased levels of both insoluble Aβ1–42 plaques and phosphorylated tau protein, which are the most relevant markers associated with the development and progression of AD [51].

In addition to impaired cognitive functions, memory, and learning, scopolamine dementia is also characterized by impaired motor coordination. Our studies also found decreased dopamine reuptake in demented experimental animals [52]. EA leads to a significant increase in dopamine levels in the brain. Dopamine reuptake is mediated primarily by dopamine transporter protein, but some nonspecific organic ion transporters are also involved in dopamine transport [53]. In animals treated with EA, a relatively small decrease in reuptake of 17% was observed, which suggests that EA most likely affects some secondary transport mechanisms. Consistent with this, experimental animals pretreated with EA showed preserved motor coordination compared to the control group. These data show another mechanism of action of EA, which has a direct bearing on the progression of NDD.

Given the general mechanisms of neurodegeneration and the pathogenesis of Parkinson’s disease (PD), a beneficial effect of EA in PD may be hypothesized. One of the factors for dopaminergic loss is represented by oxidative stress, as dopaminergic neurons are selectively susceptible to oxidative damage. The preventive effect of EA on exposure to environmental toxins is demonstrated in a model of 1-methyl-4-phenyl-1,2,3,6-tetrahydropyridine (MPTP)-induced PD [53]. Pretreatment with EA at a dose level of 10 mg/kg for one week before MPTP exposure led to lower superoxide dismutase and catalase activity compared to the control group. Increased glutathione content and decreased Cyclooxygenase-2 (COX-2) release were also observed. Therefore, EA has the potential to protect dopaminergic neurons from oxidative damage and death and plays a preventive role in the onset and progression of PD.

A previous study from our group has also established a direct relationship between levels of oxidative stress and the function of the dopaminergic neurons in the 6-hydroxydopamine (6-OHDA) rat model of PD. A direct correlation between dopaminergic system recovery and improvement in motor performance in rats was established following treatment with EA. Decrease in oxidative stress and particularly lipid peroxidation was directly associated with improved behavioral performance in EA-treated animals with PD [54].

Another study investigated the protective effects of pomegranate juice against MPTP-induced dopaminergic selective cytotoxicity and oxidative stress in human primary neurons. It is suggested that the mechanisms of this beneficial effect may be related to the antioxidant activity of phenolic constituents [16].

## 3. Protective Mechanisms of Pomegranate Polyphenols in NDD

Neurodegeneration is a multifactorial process that involves various cytotoxic mechanisms leading to neural loss. Neuroinflammation, excitotoxicity, redox-active heavy metals, high levels of free radicals, endogenous cellular defense mechanisms damage, mitochondrial dysfunction, and decreased expression of trophic factors in nervous tissue—all these processes play a role in the pathogenesis of NDD. In addition, the expression of proapoptotic proteins, which leads to neuronal death, is another key factor in the onset and progression of these diseases [55].

The well-known anti-inflammatory and antioxidant properties of ETs discussed above have attracted much interest regarding these substances as potential agents for influencing NDD.

### 3.1. Amyloid Beta Deposition

Smaller soluble Aβ oligomers play a crucial role in AD pathogenesis. Hence, selective inhibition of Aβ oligomer formation represents an optimal target for the development of innovative AD therapies.

As mentioned above, an oxidative environment can promote protein misfolding. In earlier stages, glia is involved in clearing up protein fragments and toxic oligomers of the proteins, but if the process is inefficient, as occurs with cumulative stress and ageing, misfolded proteins can influence the integrity of neuronal cells, endothelial cells and the BBB [55]. Their accumulation can lead to deficits in cognition and altered behavior. Typical examples of misfolded protein in the brain include Aβ plaques and intracellular deposits of hyperphosphorylated Tau neurofibrillary tangles. These are believed to be late markers of disease, preceded by smaller but more neurotoxic oligomers. In PD, aggregation of α-synuclein (αSyn) in Lewy bodies is also a feature. ETs appear to be able to reduce the amount of misfolded protein in the CNS not only by their antioxidant activity, which will provide a better redox balance, but apparently also by direct interaction with the proteins themselves.

Pomegranate extract limits Aβ accumulation both in vivo and in vitro with a proposed involvement of β-secretase (BACE) pathway in amyloid processing in some studies [46,47]. This was also observed in transgenic mice supplemented with EA, where Aβ decreased along with the phosphorylated forms of BACE1, Amyloid Precursor Protein (APP) and Tau. EA in vitro was able to decrease αSyn aggregation in a dose-dependent manner, to dissociate already formed aggregates and decrease their neurotoxicity. We have shown that EA is able to restore the scopolamine-induced changes in the conformation of water-soluble proteins in the brain [54,56].

PUN and EA both have the capacity to disaggregate Aβ, perhaps through their direct interaction with the hydrophobic amino acids in the amyloid structure [56]. Each of the two polyphenols appeared to produce a different organization of the amyloid fiber, and therefore may interact with different regions of this polypeptide [56]. One study revealed that EA promotes Aβ42 fibrillization and inhibits Aβ42-induced neurotoxicity [57]. A dose-dependent decrease in levels of pathogenic Aβ oligomers and Aβ cytotoxicity has also been found. This is consistent with the hypothesis that plaques are actually a protective mechanism against toxic Aβ oligomers transformed into less toxic fibrils [58].

### 3.2. Oxidative Stress

The brain is particularly susceptible to oxidative stress due to its high O_2_ consumption and low levels of endogenous antioxidants. Infection and ageing can further deplete its already low antioxidant pool [49,59]. Stress, both physical and emotional, depression and sleep deprivation can cause reactive oxygen and nitrogen species (RONS) formation at a rate that overwhelms the brain antioxidant defense [59]. Batandier et al. [60] showed that acute emotional stress can increase oxidative stress in the brain, and this is further modulated by the diet.

Oxidative stress in the CNS may lead to lipid peroxidation, which in turn could be responsible for Aβ fibrillization and neuronal death or demyelination, thus affecting the function of the brain [51].

Polyphenols are considered exogenous antioxidants that complement the endogenous antioxidant system (superoxide dismutase, catalase, glutathione peroxidase, glutathione reductase, glutathione, etc.) in order to maintain the cellular redox balance [59].

Pomegranate is able to reduce oxidized low-density lipoprotein levels and to inhibit oxidation caused by Cu^2+^ ions [9]. Pomegranate polyphenols and their metabolites can decrease lipid peroxidation in the nervous system in a variety of RONS-generating experimental setups. This effect is unlikely to be caused by urolithin metabolites of pomegranate, which showed 42-fold lower antioxidant activity than PUN [17]. 

Aqueous pomegranate extract had a protective effect against reactive oxygen species ROS production in brains, and simultaneously decreased lipid peroxidation [38]. The extract, along with purified PUN, was able to trap the OH radical and ONOO^−^, in contrast to EA at the same concentrations. EA was also less effective at interacting with superoxide, but better at protecting against lipid peroxidation. PUN decreased lipid peroxidation after brain hemorrhage, as well as after ischemic stroke. Pomegranate extract prevented lipid peroxidation in the hypothalamus of spontaneously hypertensive rats [61]. In the hippocampus, pomegranate flower extract also decreased oxidized lipids. In the same brain region, in mice under chronic stress, pomegranate fruit extract decreased lipid peroxidation, as measured by malonyl dialdehyde (MDA). Pomegranate peel extract lowered oxidized lipids and NO (nitric oxide) accumulation not only in animals treated with oxidative stress-inducing aluminum, but also in untreated animals [35]. This change in the lipidome of healthy animals may be advantageous if they are subsequently exposed to oxidative stress.

As noted above, EA appears to be particularly efficient at preventing lipid oxidation. This was shown in our studies on a rat PD model and on a mouse scopolamine-induced dementia [35,56], in the hippocampus of sleep deprived animals [30]. EA acted on a subcellular level in a Huntington’s disease model, decreasing lipid peroxidation in brain mitochondria [62]. A specific property of UA but not EA is the stimulation of sphingolipid synthesis from palmitic acid, in oligodendrocytes [63]. In these cells, EA was also able to preserve demyelination but did not stimulate sphingolipid synthesis [64]. Many studies on the effect of pomegranate polyphenol supplementation have also measured either the expression levels or activity of the endogenous antioxidant enzymes: superoxide dismutase (SOD), catalase (CAT), glutathione peroxidase (GPx), glutathione reductase (GR) and glutathione (GSH). A study conducted by our group demonstrated that EA improved the antioxidant capacity of neurons in the 6-OHDA model of PD by increasing CAT, SOD and GSH activity [56]. However, unlike the case of decreased lipid peroxidation, healthy animals did not experience upregulation of antioxidant enzymes SOD, CAT, GPx, and GR when exposed to pomegranate polyphenols. 

The beneficial effect of pomegranate extracts and active compounds seems to decrease at high concentrations, under which conditions the polyphenols may act as prooxidant [65]. Increased activity or expression of the endogenous antioxidant enzymes was observed in a variety of stress-inducing conditions [66,67,68,69] and brain regions, as well as in vitro, indicating a direct effect on neuronal cells. Even though similar effects were achieved for manganese-treated neurons with other well-known antioxidants, such as vitamin E and niacin, the greatest effect was observed with PUN [54]. This ET changed intracellular antioxidant levels dose dependently in human neuroblastoma culture [69], showing that pomegranate-derived substances modulate not only the antioxidant capacity of the extracellular milieu, but also intracellular redox balance.

In the process of respiration, mitochondria produce semi-reduced free radicals, superoxide and hydrogen peroxide, that cause oxidative stress [66,67,68,69,70,71]. In NDD, various oxidative reactions lead to neuronal death [72], and molecules such as vitamins, lipoic acid, and antioxidant enzymes, as well as redox-sensitive protein transcription factors, are able to alleviate the neuronal oxidative stress [73,74,75,76,77].

A growing body of data documents the neuroprotection exerted by EA in preclinical models. In a model of Iron (II) sulfate toxicity on PC12 cells, EA demonstrated a strong antioxidant effect by reducing the levels of the endogenous cellular antioxidant GSH. EA both influenced the expression and increased GSH levels, thus exerting its antioxidant activity. Because of its chelating properties, EA also counteracts iron- and copper-induced ROS formation [78].

EA administration in 2,3,7,8-Tetrachlorodibenzo-p-dioxin (TCDD)-challenged rats exerted protective effects on cortex and hippocampus via a significant reduction in superoxide anion production in these brain regions and by inducing GSH synthesis and increasing glutathione peroxidase (GPx) activity. Additionally, a significant decrease in thiobarbituric acid reactive substances (TBARS) was observed, as well as a significant reduction in TCDD-induced DNA damage. TCDD is known to form a complex that directly alters the DNA, which is why the authors hypothesize that EA has the capacity to bind to DNA and prevent TCDD from interacting with it [79].

Based on the documented neuroprotective effects of EA, it can be hypothesized that EA may have therapeutic potential to influence the progression of NDD, such as AD and PD, as their main feature is neuronal loss.

### 3.3. Inflammation

There is a vast amount of data confirming the role of inflammation in NDD, including AD and PD. Chronic inflammation can lead to neuronal loss, and the activation of microglia can contribute to the onset and progression of NDD including AD, PD, Amyotrophic Lateral Sclerosis (ALS) and MS. When macrophages are classically activated to M1 polarization, they induce a proinflammatory environment, while a shift to an M2 profile promotes neuroprotection [80,81]. UA is able to induce M2 polarization in bone marrow-derived macrophages (BMDM) and microglia, showing that it can elicit similar responses in both peripheral and resident immune cells [80,81].

In peripheral macrophages, punicalin had the strongest effect in shifting the profile away from M1, stronger than pomegranate juice, EA or gallic acid [81]. UA, EA, and pomegranate flower extract decreased Glial fibrillary acidic protein (GFAP), a marker of astroglia activation. In a model of PD, both astrocyte and microglia activation were suppressed by UA [82] The EA effect was particularly strong in the astroglia-neuron, but not in the microglia-neuron coculture, even without direct contact between the different cell types [83]. 

Three proinflammatory cytokines, IL-1β, IL-6 and TNFα, were increased in response to multiple stressors but decreased in brain tissue in vivo and in vitro upon administration of pomegranate extracts and ET downstream metabolites [84,85,86,87]. EA has also been shown to affect neuroinflammation by suppressing the NLRP3 inflammasome, involved in the neuroinflammation process in PD. In in vitro studies, EA has been found to inhibit nigrostriatal dopaminergic neurons specifically by affecting NLRP3 function [88].

The levels of the anti-inflammatory IL-10 were also investigated, and were found to increase in parallel with the decrease in proinflammatory cytokines [46,61]. In some reports, the cytokine levels in serum changed as well, indicating a systemic inflammatory process not limited to the CNS, which nevertheless responded to the presence of pomegranate polyphenols [46,61].

IL-17 is a cytokine produced by Th17 cells that may be implicated in the pathogenesis of MS. It also mediates monocyte–endothelial interactions and passage through the BBB. Pomegranate extract dose-dependently suppressed IL-17 generation in CD4+ activated cells, without altering IL-17 levels in healthy rats [85]. Additionally, UA has been shown to decrease IL-17 production in and microglia [46].

Prostaglandins (e.g., PGE2), produced by cyclooxygenase (COX), contribute to the development of inflammation and pain perception. They may also be involved in creating an inflammatory environment that stimulates protein misfolding in AD [86]. Both amyloid formation and PGE2 synthesis in neuroblastoma cells stimulated with IL-1β were lowered upon the addition of pomegranate extract [86]. COX2 decreased with PUN treatment in manganese-induced stress [54]. Furthermore, PUN decreased not only COX2, but also mPGES1, as well as PGE2 levels in the supernatants of microglia cell culture and hippocampal slice culture [83]. Thus, pomegranate ETs are able to modulate prostaglandin synthesis in several CNS cell types.

It is likely that EA interacts with the Fe in the protoporphyrin active site of COX enzymes. This hypothesis is supported by the lack of an effect when competitive COX inhibitors are used in conjunction with EA treatment [87]. The effect is strongly dose dependent, with a bell-shaped curve of different concentrations of EA on PGE2 synthesis. Up to 1 µM EA, PGE2 synthesis was stimulated, and above 10 µM, it was slightly decreased [87]. At an oral dose of 6 mg/kg, EA also increased [PGE]_plasma_ in rats [87].

Autophagy is linked to neuroinflammation and neurodegeneration [89]. Inefficient autophagy leads to the accumulation of debris, which in turn leads to apoptosis [79]. In chronic inflammation, it has been hypothesized that M2 polarization cannot be maintained due to insufficient or ineffective mitophagy and autophagosome flux. Both PUN and EA induce autophagy and improve autophagosome formation [81]. Toney et al. [90] reviewed the role of UA in different pathological scenarios, including neuropathology, and showed that UA is able to improve autophagy during inflammation and consequently, to increase cell viability, via the regulation of the mechanistic target of rapamycin (mTOR) pathway [91]. UA increased autophagy in microglia and its neuroprotective effects were lost when autophagy was blocked [89]. UA also increased autophagy in neurons following trauma [92].

Sun et al. observed that the regulator of mitophagy, Beclin1, was affected by exposure to pomegranate extract. This maintained mitochondrial clearance and mitochondrial biogenesis at appropriate levels [61]. Mitophagy was more effective when UA pretreatment was given to lipopolysaccharide (LPS)-stimulated microglia. When a mitophagy inhibitor was used, the UA suppression of NLRP3 inflammasome activation was abolished, suggesting that the urolithin acts by supporting the mitophagy flux in microglia [93]. If mitophagy is inefficient, ROS generation from mitochondria increases and can activate the inflammasome NLRP3, thereby linking oxidative stress and inflammation [94].

Whole pomegranate extracts, PUN, EA, and UA, administered by different routes in vivo, as well as in in vitro studies, revealed that such treatment decreases the pro-apoptotic Cas-3 and Bax and increases the antiapoptotic Bcl-2 at both the transcript and protein levels in neurons, microglia and oligodendrocytes.

From the above, it may seem that an antiapoptotic effect, such as the one observed for CNS tissues and stressed neuronal and glial cells is the default response to pomegranate ETs. However, Venusova et al. [94] reviewed the immune and physiological functions of PUN, which in cancer cells increased Bax and decreased Bcl-2 and Bcl-XL to stimulate apoptosis, release of mitochondrial cytochrome c, and activation of Cas-9 and Cas-3. In Herpes simplex virus 1 (HSV-1)-infected microglia, the ET corilagin had a pro-apoptotic effect [12]. This occurred despite the parallel anti-inflammatory response that it elicited (lower TNFα, NO and IL-1β) and showed that ETs act on apoptosis selectively and in concert with other signals to achieve a response relevant to the current stress that cells are experiencing.

### 3.4. Neurogenesis

Neurogenesis is a process that occurs in several regions of the healthy mature brain: the olfactory bulbs, the ventricular region, and the dentate gyrus of the hippocampus. Neurogenesis is a multistep process that includes proliferation, migration, differentiation, and maturation, with the final step of synapse formation [95]. Mood disorders, chronic stress, and neurodegeneration may reduce neurogenesis. A measure of neurogenesis is represented by the levels of BDNF. This molecule is active in the hippocampus, cortex, and basal nuclei of the forebrain and contributes to cognitive performance, learning and memory. BDNF levels increase following antidepressant treatment and are depleted during chronic stress [37].

Research studies on AD have shown a link between impaired neurogenesis and neurodegeneration, but the interrelated factors underlying the two processes are not fully understood [95]. Modulation of neurogenesis could be considered a potential therapeutic approach for the treatment of AD and other NDD. In a study conducted by Tabopda et al. on neural stem cells, EA demonstrated an effect on neurodegeneration by a not fully understood mechanism [96]. The authors used methanolic extracts of tree bark and EA, with two EA derivatives isolated (3,3′di-O-methylellagic acid and 3,3′-di-O-methylellagic acid-4-O-beta-D-xylopyranoside). Both showed potential to induce neuronal differentiation without cytotoxic effect. This makes the derivatives potential candidates for pharmacological agents. 

### 3.5. Blood–Brain Barrier Integrity

The BBB is a system of interacting cells including neurons, astrocytes, pericytes, and endothelial cells [97]. In order for the pomegranate ETs to interact directly with misfolded proteins in vivo, they need to access the brain tissue from the systemic circulation. EA and its metabolites cross the blood–brain barrier, which is confirmed by several studies demonstrating the effects of EA on the CNS. Indeed, isotopically labeled EA was identified in the brain of mice after i.p. administration at very low concentrations of 5–8 nmol/g [98]. In another study, EA appeared in the brain at the ng/g level within 0.5–4 h after a single dose of 50 mg/kg orally [30]. Kujawska et al. [56] measured UA at 1.68 ng/g tissue in the brain, about 10-fold less than that in the plasma. This confirmed that metabolites from the intragastric administration of pomegranate juice were able to eventually reach the brain. In an in vitro transwell culture of neuroblastoma, endothelial and astrocyte cells, PUN and EA were able to pass through the model barrier with more EA passing through compared to PUN [80].

The urolithins (the active metabolites of EA) are considered lipophilic enough to be able to cross biological barriers, and low-molecular-weight polyphenols are more bioavailable in general [97,98,99]. However, the BBB is less permeable to sulfate and glucuronide conjugates [97]. This may account for the varying activity of urolithins and their conjugates. For example, Urolithin A (UA) and its methylated conjugate (mUA) had the strongest neuroprotective effect on differentiated neurons after coculture with activated microglia. Conjugated urolithins were more active in the inhibition of nuclear factor kappa B (NFκB), an important regulator of inflammation and oxidative stress, while free UA was more efficient at inhibiting the proinflammatory TNFα production and reducing iNOS expression [99]. The response of activated microglia to urolithins and their methyl-conjugates was also explored by Xu et al. [82] and free UA was found to be the most potent.

One possibility is that inflammation increases the leakiness of the BBB, allowing larger polyphenols to pass through more easily [97]. This would explain why pomegranate polyphenols have an effect on CNS during inflammation or oxidative stress, but exert no or limited effects on a healthy CNS.

A key player in determining the availability of free UA and other low molecular weight polyphenol metabolites in the CNS may be the activity of β-glucuronidase, released from neutrophil granules at the site of inflammation [99]. This enzyme hydrolyses less active conjugates to their free form [100,101,102]. A study showed that LPS stimulation of a pro-inflammatory response increased deconjugation of UA-glucuronide in several organs (liver, lung, spleen, and bladder), and thus increased the concentration of free UA in organs. On the other hand, higher levels of UA-glucuronide were detected in plasma and may serve to increase UA delivery to inflammatory hotspots in the body [102].

The urolithins are also capable of preventing the release of granules from primary human neutrophils [99]. This could serve as a negative feedback response, controlling neutrophil degranulation in inflamed tissue and limiting free UA production when concentrations of the deconjugated metabolite are already high in situ.

Once present in their bioactive form in the brain, free urolithins may limit inflammation by improving the integrity of the BBB. For example, after traumatic brain injury, UA was able to reduce BBB permeability, increase the expression of tight junction proteins and close gaps in the barrier that formed after the injury [89]. In a model of intracerebral hemorrhage in rats, PUN treatment reduced the infiltration of immune cells and improved barrier function. This occurred only when the brain was experiencing injury and not in healthy animals [63]. Barrier function was also improved after ischemic stroke where PUN was used as pretreatment [62]. Similarly, corilagin was effective in preventing infiltration of inflammatory cells and interstitial edema in HSV-1 infected mice brains [12]. UA was also effective at preventing infiltration of dendritic cells and Th1/ Th17 cells into the CNS in a MS model [45]. EA did not prevent the infiltration of perivascular spaces with immune cells or the number of activated microglia, in an experimental autoimmune encephalitis (EAE) model, but in another study, EA reduced CD45 levels in the brain in both young and old animals, used as a measure for the amount of infiltrating immune cells. Animals treated with pomegranate peel extract and the oxidative-stress-generating aluminum had lower levels of aluminum in the brain [8]. This may be indicative of a better barrier function, or, as the authors hypothesized, a complex forming between the large ET PUN and the metal ions, making them too big to pass through the BBB. The improved barrier function by pomegranate polyphenols is not specific to the BBB and in the study by Singh et al. [103], gut barrier integrity was improved though reduced permeability and increased tight junctions.

### 3.6. Neurotransmitter Interactions

The proper functioning of the CNS depends on a fine balance of correct synthesis, sensing, recycling, and removal of neurotransmitters by neurons and glia. Disturbances in this process can lead to a decline in mental health and CNS function [104]. The pomegranate ETs have been shown to affect the levels of a multitude of neurotransmitters. This is not a universal effect of ETs, since depending on the experimental setup and the stress induced in the CNS, one or more neurotransmitters may be affected, while the others remain unchanged.

We demonstrated that in a rat model of PD, dopamine (DA) levels were restored by pretreatment with EA, and UA was able to protect the dopaminergic neurons in vivo [54]. Importantly, EA does not seem to directly protect dopaminergic neurons from 6-OHDA toxicity in vitro, even though oral gavage in vivo prevented dopaminergic loss in the striatum [54]. In a study of scopolamine-induced dementia by Tancheva et al. [34], we showed that DA uptake increased by oral EA supplementation. This protection may be driven by astroglia, as shown by Wei et al. [105]. DA released in the extracellular space can lead to oxidative stress, and can be neurotoxic if not controlled. In astroglia, oxidative stress activates Nrf2 signaling, which releases GSH to support antioxidant levels for neurons [105].

Cholinergic neurons, important for memory and learning, also appeared to be subject to pomegranate polyphenol protection. One of the typical hallmarks of AD is the loss of cholinergic neurons, along with the accumulation of beta-amyloid and hyperphosphorylated tau protein [106,107,108], as well as the increased activity of enzymes that hydrolyze acetylcholine in the synapses of cholinergic neurons—butyrylcholinesterase and acetylcholinesterase [109]. This pathogenetic mechanism of AD has been studied since the 1980s [110,111], and resulted in the introduction of cholinesterase inhibitors as the first class of medicinal products for the treatment of AD, led by the plant-derived galantamin. Oral supplementation of EA in mice with scopolamine-induced dementia (to mimic AD) showed that this pomegranate metabolite reduced AChE activity. Methylated conjugates of EA appeared more effective in inhibiting AChE than EA itself, acting as reversible competitive inhibitors. The same effect was observed when galactose-induced ageing was induced in mice in the presence of UA, during manganese treatment accompanied by PUN to induce PD-like symptoms, and with pomegranate juice to counter aluminum toxicity [33,105,112,113].

Long-term use of cholinesterase inhibitors is associated with a series of adverse effects [114], necessitating the search for other therapeutic approaches that affect the cholinergic signal. Potential therapeutic candidates that meet this requirement are the phenolic acids, with EA being a representative of this group. EA as a single substance, however, does not show significant cholinesterase activity in vitro [115]. On the other hand, EA-rich extracts and derivatives, such as walnut (*Juglans regia*) extract, show pronounced in vivo cholinolytic effects [116,117].

The currently available in vitro and in vivo data suggest that EA alone most likely does not possess cholinolytic activity. On the other hand, EA and the ETs found in natural extracts after administration are converted into their biologically active metabolites, urolithins, which exert anticholinesterase activity, as demonstrated by Norouzbahari and co-authors [118].

The levels of the amino acid glutamate (Glu) in sleep deprivation increased and correlated with ROS production and loss of neuronal viability. Treatment of primary hippocampal neurons with EA made them more resilient to Glu exposure and improved their viability in an Nrf2-dependent process [29]. Pretreatment of hippocampal neurons with PUN also seems to inhibit Glu-induced excitotoxicity [105] and restore the Glu/GABA balance in the striatum following manganese exposure [53].

In depression, the levels of monoamines are lowered. EA improved depressive behavior in mice, and this appeared to be dependent on noradrenergic and serotonergic systems, in both stressed and unstressed mice [32,36,37]. Supplementation with pomegranate juice for a year influenced tryptophan and downstream serotonin metabolism [119]. The improvement in depressive behavior in ovariectomized rats was mediated both by the estrogen receptor ERβ, which is responsible for the mood-regulating role of estrogens, and via the serotonergic system [38]. Serotonergic neurons express ERβ receptors, which regulate the rate-limiting step of the conversion of tryptophan to serotonin (tryptophan hydroxylase, TPH). Given that the animals were treated with a complex pomegranate extract, the authors provided a list of pomegranate compounds besides the ETs, which may have synergistic antidepressant activity. Pomegranate-derived compounds may therefore be beneficial for women, who are especially vulnerable to depression during estrogen fluctuations such as in perimenopause [39]. Interestingly, UA was able to interact with another steroid molecule, 1,25-dihydroxyvitamin D3 and enhanced the transcriptional regulation of TPH2 in rat serotonergic raphe cell line. This translated to higher levels of serotonin being secreted in the presence of UA in the cell culture media [39].

Proinflammatory cytokines can activate indoleamine-2,3-dioxygenase (IDO), which affects tryptophan catabolism, therefore inhibiting its conversion to serotonin downstream, with a consequent reduction in serotonin levels. The levels of IDO were reduced when mice under chronic stress exhibiting anxiety and depressive behavior were given high doses of pomegranate extract. The hypothalamus histology returned to normal and serotonin in this brain region increased [120]. These effects may be due to an upstream inhibition of an inflammatory response and the associated reduction of proinflammatory cytokines by pomegranate polyphenols. Thus, limiting neuroinflammation is one possible pathway by which ETs regulate neurotransmitter levels.

5HT receptors and α1- and α2-adrenergic receptors appeared to be involved in the antidepressant effects of EA [27]. Noradrenaline exocytosis in cortical synaptosomes could be stimulated in old mice treated with EA for 2 weeks. Noradrenaline and adrenaline levels were lowered after pomegranate extract treatment in mice under chronic mild stress and corresponded to Monoamine oxidases (MAO)-B activity responding to the pomegranate polyphenols [31]. The levels of both dopamine and serotonin were restored with pomegranate juice in aluminum toxicity [113,121]. 5HT returned to normal values, as did DA and noradrenaline in the striatum of rodent brains of a PD model, when they were fed PUN prior to exposure to manganese [53]. The effects of ETs on multiple receptors suggest that the ETs do not act on a monoamine neurotransmitter-specific level but rather prevent upstream stress signals that may cause neurotransmitter imbalance.

The anxiolytic effect of EA in mice appeared to be mediated by the inhibition of the serotonergic system for depression, but also relied on GABA-ergic neurotransmission [27,28]. Gamma-aminobutyric acid (GABA) can be influenced by different flavonoids and contribute to their benzodiazepine-like anxiolytic effects [28]. This appeared to be driven by binding to the benzodiazepine site of GABA A receptors. BDNF can also modulate neuronal excitability levels by inhibiting GABAergic mediation and its increased levels after EA administration can indirectly affect the GABA balance [122].

The above results reveal the complexity of ETs as neuroprotective agents and make them potential candidates for preventive and therapeutic agent, which would have the ability to influence the multifactorial pathogenesis of NDD by a multi-targeted mechanism.

## 4. Conclusions

The multitude of in vitro and in vivo studies summarized in the present review suggests that pomegranate polyphenols act on both neuronal and glial cells, and also affect BBB function, restoring redox balance in the blood and brain and increasing blood flow to the brain.

Recently, the interest in these molecules as potential agents for the treatment of NDD has increased. Independent studies have demonstrated that the significant neuroprotective effects of ETs are mediated by their antioxidant and anti-inflammatory effects, chelating properties, by their ability to activate various signaling pathways, as well as the ability to influence mitochondrial damage, thus regulating autophagy, apoptosis, and neurotransmitter signaling.

Importantly, complex pomegranate extracts have more pronounced effects, as described above, in comparison to purified components such as EA, PUN, etc. Last but not least, based on the efficiency of ETs’ active metabolites, the urolithins, oral administration is a feasible route for the administration of polyphenol-rich pomegranate products, and hence they may be used as dietary supplements.

## Figures and Tables

**Table 1:** Pomegranate products commonly used in research studies.

Condition	Experimental Model	Type of Extract
AD	APPsw/Tg2576	pomegranate extract
	APP/PS mice	ellagic acid
	scopolamine induced ICR mice	ellagic acid
	HT22 mice hippocampal neurons	punicalagin
	BBB model triculture-astrocyte, neuron, endothelial	punicalagin, ellagic acid
PD	C57BL/6 mice rotenone induced	ellagic acid
	Sprague-Dawley rat MnCl_2_-induced	punicalagin
	α-syn expressing SHSY5Y cells	ellagic acid
	MPTP-treated primary neurons	pomegranate juice extract
	6-hydroxydopamine challenged SH-SY5Y cells	punicalagin
	MPTP-treated mice	urolithin
Huntington’s disease	adult Wistar rats 3-nitropropionic acid treated	ellagic acid
MS	Lewis rats EAE	ellagic acid
	rat C6 astroglia, human HOG oligodendrocytes	ellagic acid, urolithin A or B
	LPS-stimulated SIM-A9 microglia	urolithin A
	BV2 murine microglia	urolithin A
	C57BL/6 mice EAE	urolithin A
D-galactose induced ageing	ICR mice; Sprague-Dawley rat	urolithin A; ellagic acid
ischemia/reperfusion	mouse neuroblastoma N2a cells	urolithin A
	Wistar rats by middle cerebral artery occlusion	punicalagin
Oxidative stress	PC12 cell induced by H2O2	punicalagin
	BV2 murine microglia; human SH-SY5Y neurons; induced by H2O2	urolithins
	Wistar rates female induced by Al	pomegranate peel extract
	SWR/J mice female induced by Al	pomegranate juice
	rat primary neuron culture induced by cisplatin	pomegranate peel extract
	Wistar rats induced by Cu-nanoparticle exposure	pomegranate juice
	Sprague-Dawley rat microglia primary culture LPS challenged	punicalagin
	C57BL/6 mice hippocampal slice culture LPS challenged	punicalagin
	BV2 murine microglia LPS challenged	urolithins
	Wistar rats LPS challenged	ellagic acid
	SK-N-SH cells IL1b stimualted	freeze-dried pomegranate
Depression/anxiety	Sleep deprivation C57BL/6J mice; Wistar rat	ellagic acid; punicalagin
	chronic mild stress in C57BL/6 mice	pomegranate peel extract; ellagic acid
	Induced menopause in female Wistar rats	ellagic acid; punicalagin; pomegranate extract
nociception	adult Wistar rats- radiant heat exposure	ellagic acid; pomegranate peel extract
	Swiss mice-acetic acid stimulation	ellagic acid
	albino mice-formalin or acetic acid	pomegranate peel extract
	Wistar rats sciatic pain	punicalagin
HSV-1 encephalitis	BV-2 microglia; Balb/c mice	corilagin

## Data Availability

Not applicable.

## Abbreviations

6-OHDA: 6-hydroxydopamine; AChE: acetylcholinesterase; AD: Alzheimer’s Disease; ALS: Amyotrophic Lateral Sclerosis; APP: Amyloid Precursor Protein; BACE: β-secretase; BBB: Blood–brain barrier; BDNF: Brain-Derived Neurotrophic Factor; BMDM: Bone Marrow-Derived Macrophages; CAT: Catalase; CNS: Central Nervous System; COX-2: Cyclooxygenase-2; DA: Dopamine; EA: Ellagic acid; ETs: Ellagitannins; fMRI: Functional Magnetic Resonance Imaging; GABA: Gamma-aminobutyric acid; GFAP: Glial fibrillary acidic protein; Glu: Glutamate; GPx: Glutathione Peroxidase; GR: Glutathione Reductase; GSH: Glutathione; HSV-1: Herpes simplex virus 1; i.p.: intraperitoneal; ICR: Institute of Cancer Research; IL-1β: Interleukin-1β; IL-6: Interleukin-6; LPS: Lipopolysaccharide; MAO: Monoamine oxidases; MDA: malonyl dialdehyde; MPTP: 1-methyl-4-phenyl-1,2,3,6-tetrahydropyridine; MS: Multiple Sclerosis; mTOR: Mechanistic Target Of Rapamycin; NDD: Neurodegenerative diseases; NFκB: Nuclear Factor kappa B; NLR: Nod-like receptor; NLRP3: NLR family of proteins pyrin domain containing 3; NO: Nitric Oxide; p.o.: per os; PD: Parkinson’s disease; PUN: punicalagin; RNS: reactive nitrogen species; RONS: Reactive Oxygen and Nitrogen Species; ROS reactive oxygen species; SOD: Superoxide Dismutase; TBARS: thiobarbituric acid reactive substances; TCDD: 2,3,7,8-Tetrachlorodibenzo-p-dioxin; TNF-α: Tumor Necrosis Factor-α;TPH: Tryptophan Hydroxylase; UA: Urolithin A.
